# Preoperative anxiety among adult patients undergoing elective surgery: a prospective survey at a general hospital in Ethiopia

**DOI:** 10.1186/s13037-019-0198-0

**Published:** 2019-04-08

**Authors:** Asres Bedaso, Mohammed Ayalew

**Affiliations:** Hawassa University, College of medicine and health sciences, School of Nursing, Hawassa, SNNPR Ethiopia

**Keywords:** Anxiety, Preoperative, Surgery, Yirgalem, Ethiopia

## Abstract

**Background:**

Major life changes are among factors that cause anxiety, and one of these changes is surgery. Hospitalization, regardless of disease, is known to provoke anxiety in the patient admitted for surgery. Anxiety is an unpleasant disturbing experience that involves way of thinking of tension, apprehension, uneasiness and high autonomic activity. Patients with high levels of anxiety require higher doses of anesthetic induction agents and recover poorly. The objective of this study was to investigate the prevalence of preoperative anxiety and its predictors among adult patients scheduled for elective surgery.

**Methodology:**

Institutional based cross sectional study was conducted using interviewer administered structured questionnaire in Yirgalem zonal hospital in Ethiopia from November 1, to December 30, 2018 on 407 patients scheduled for elective surgery. The study included all patients with age greater than 18 years who were undergoing surgery. Patients with known anxiety disorder and unable to communicate were excluded from the study. State and trait anxiety inventory (STAI) measurement scale was used to assess preoperative anxiety. Statistical analysis was performed using SPSS version 22. Binary logistic regression analysis was performed to determine the predictors of preoperative anxiety. The strength of the association was presented using AOR with 95% confidence interval and *p*-value < 0.05 was considered as statistically significant.

**Results:**

Among a total of 402 patients enrolled in the study 228 (56.7%) were male. The prevalence of preoperative anxiety among scheduled patients for elective surgery was 47.0%. Having strong social support (AOR = .16 CI = 0.07, 0.34), harm from doctor or nurse mistake (AOR = 5.03, CI = 2.85, 8.89), unexpected result of operation (AOR = 3.03, CI = 1.73, 5.19), unable to recover (AOR = 2.96, CI = 1.18, 4.87), and need of blood transfusion (AOR = 2.76, CI = 1.65, 4.62) were significantly associated with preoperative anxiety.

**Conclusion:**

In the current study the prevalence of preoperative anxiety was high (47%). Having strong social support, unexpected result of operation, harm from doctor or nurse mistake, need of blood transfusion, and unable to recover were found to be statistically significant for preoperative anxiety. Patients need to be assessed regularly for anxiety during the preoperative visit.

**Electronic supplementary material:**

The online version of this article (10.1186/s13037-019-0198-0) contains supplementary material, which is available to authorized users.

## Background

Anxiety can be defined as feelings of tension, uneasiness, nervousness, fear and high autonomic activity with varying degree of intensity. Surgery is a traumatic treatment that usually associated with bleeding, pain, the risk of morbidity or sometimes death. Anxiety is disagreeable emotional experience which may cause patients undergoing intended surgical operation to avoid such procedures [1]. Efforts at assessing and reducing preoperative anxiety should include timely preoperative visit by the anesthesiologist, and appropriate premedication and psychological preparation of the patient [2].

Preoperative period is worrying events that generate specific emotional, cognitive, and physiological responses of a patient [3, 4]. Surgery-related anxiety is somewhat commonly accepted as a normal reaction in pre-operative patients. Research has established that waiting for surgery or invasive procedures is stressful and anxiety aggravates and affects both physiological and psychological parameters [5].

Preoperative anxiety has a great influence on the surgery outcomes. It could cause hypertension, increase heart rate, and thus, might lead to bleeding. Besides, it has been shown that high level of preoperative anxiety is correlated with an increased postoperative pain-relieving requirement [6]. Anxiety is a personality feature of responding to certain situations with a stress syndrome of responses. Anxiety states are then a function of the situations that evoke them and the individual personality that is prone to stress [7]. All of the features of Anxiety results in cognitive impairment characterized by impaired thinking, decision making, perception and concentration [8, 9].

Major life changes are among factors that cause anxiety, and one of these changes is surgery. Hospitalization, regardless of disease, is known to provoke anxiety in the patient admitted for surgery. Patients with high levels of anxiety require higher doses of anesthetic induction agents and recover poorly. If unrecognized, prolonged anxiety creates stress which may subsequently harm the patient and delay recovery [10].

The degree to which each patient manifests anxiety related to future experiences depends on many factors. These include age, gender, type and extent of the proposed surgery, previous surgical experience, and personal susceptibility to stressful situations [11]. Multiple recent studies have investigated the association between preoperative anxiety and morbidity/mortality rates. These studies declared that preoperative anxiety is self-sufficient predictor of postoperative morbidity and mortality including late mortality using survival analysis [8].

The morbidity and mortality related with anxiety is more significant in aged and cardiac patients [9]. All of these conditions have cost implications for patients, hospitals and countries. These also affect health care outcomes and patient satisfaction [9, 10]. White coat hypertension which is commonly associated with anxiety occurred in medical settings. It is not benign phenomenon. Similarly with undiagnosed or uncontrolled hypertension, it can cause glucose intolerance, organ damage, cardiac morbidities and mortalities [11]. Anxiety is also one of the possible causes for unnecessary operation cancelations. The overall prevalence of preoperative anxiety as reported in some studies is in range of 60–80% in western population but some researchers showed a wider range, which is 11–80% [12, 13].

Therefore, the objective of this study was to determine the prevalence of preoperative anxiety and its predictors among adult patients scheduled for elective surgery.

## Methods and material

### Study design, area and period

Institutional based cross-sectional study was employed from November 1, to December 30, 2018. Study participants were recruited consecutively until the estimated sample size fulfilled. The study was conducted at Yirgalem general hospital, which is found in Yirgalem town. The hospital is located south of the national capital Addis Ababa and southeast of regional capital Hawassa. It served about 65,222 patients in 2017. The hospitals provide different services like surgical service, medical, Obstetric and gynecological, pediatric, orthopedic and Emergency. The numbers of patients who undergo surgical procedure in each unit differ according to the extent of procedure to be done, flow of patient, availability and function of surgical equipment. The hospital provides a number of surgical therapies for the patient such as, major surgery, minor surgery, obstetric and gynecological surgery etc.

### Source and study population

The source populations were all patients diagnosed and scheduled for elective surgery in Yirgalem general hospital. The study population consisted of adult patients scheduled for elective surgery at Yirgalem general hospital that were available during data collection period.

### Study unit

The study unit was individual patient diagnosed and scheduled for elective surgery in Yirgalem general hospital.

### Inclusion and exclusion criteria

The study included all adult patients with age greater than 18 years who were scheduled for elective surgery during the study period. Patients with known anxiety disorder, unable to communicate and took any type of anxiolytics were excluded from the study.

### Sample size determination and sampling technique

Single population proportion formula was used to calculate the sample size. Prevalence of preoperative anxiety 59.6% was taken from study done in Gondar hospital, northwest Ethiopia 2017 [8]. Also, 95% CI and 5% marginal error were the assumptions considered.

*n* = 370 surgical patents, adding 10% non-response rate, the final sample size will be 407.

### Data collection instrument

The data collection tool for the study has four parts. The first part of the tool contains questions on socio-demographic and clinical factors, the second part contains questions to asses social support of study participants, the third part assess the possible causes of preoperative anxiety and the fourth part is state-trait anxiety inventory (questionnaires): STAI Form Y-1 and STAI Form Y-2 questionnaire (Additional file 1).

Data collection tools on preoperative anxiety were adapted and modified from validated questionnaire used on other study [11]. The questions and statements were grouped and arranged according to the particular objectives that it can address based on experts comments.

Level of anxiety and need for information about surgery and/or anesthesia were assessed with the state trait anxiety inventory scale (STAI). The STAI was suitable for individuals who are greater than 18 years old. The STAI-Y Form is the definitive instrument for measuring anxiety in adults. It clearly differentiates between the temporary condition of “state anxiety” and the more general and long-standing quality of “trait anxiety”. The STAI has 40 questions with a range of four possible responses to each.

The State Anxiety Scale (STAI Form Y-1) consists of 20 statements that evaluate how the respondent feels “right now, at this moment”. The Trait Anxiety scale (STAI Form Y-2) consists of 20 statements that evaluate how the respondent feels “generally”. In responding to the STAI-Anxiety scale, the subjects choose the number that best describes the intensity of their feelings: (1) Not at all, (2) somewhat, (3) moderately and (4) very much. In responding to the T-Anxiety scale, subjects rate the frequency of their feelings on the following four-point scale: (1) almost never, (2) sometimes, (3) often and (4) almost always. Scores for both the S-Anxiety and the T-Anxiety scales can vary from a minimum of 20 to a maximum of 80. The sum of the scores on all items constitutes the individual’s score [11].

### Data collection technique

Patients who were diagnosed and scheduled for elective surgery interviewed using interviewer administered technique. The data was collected by two trained psychiatry nurses.

### Data quality control measurement

The data collection tool was pre-tested on 5% of patients who were diagnosed and scheduled for elective surgery in Adare hospital to check the clarity of the tool and identify any confusing or any vague questions. The data collection was done under close supervision by research team. Completeness of data was checked daily and coded before data entry.

### Data analysis

The completed questionnaires were checked for inconsistencies and missed values. Incomplete questionnaires were excluded from the analysis. Before data entry, appropriate coding and editing was performed. After data entries checked, the analysis was performed using SPSS version 22 software. Descriptive statics such as percentage and frequency distribution for different characteristics was used in data. Common descriptive statistics were considered as per variables of interest. Bivariate and multivariate logistic regression analysis was performed. The strength of the association was presented using AOR with 95% confidence interval and *p*-value < 0.05 was considered as statistically significant.

### Operational definition

Anxiety: is state of feeling of an unlikable disturbing experience of the respondents with STAI score of > 44 and above, and those who scored less than 44 don’t have preoperative anxiety. Substance use: is improper usage of any type of psychoactive chemicals within the last 3 months.

## Results

### Socio-economic and demographic characteristics

A total of 402 patients were incorporated in this study with 98.77% response rate. The study enrolled 228 (56.7%) male, 167 (41.5%) were Protestant by religion, 292(72.6%) of the respondents were married and majority of the participants 213(53%) came from urban (Table 1).

**Table 1:** Socio-demographic characteristic of adult patient undergoing elective surgery at general hospital in Ethiopia, 2018 (*n* = 402)

Variables	Categories	Frequency(*N*)	Percentage
Sex of respondents	Male	228	56.7
	Female	174	43.3
Age of respondents	18–30	50	12.4
	31–45	146	36.3
	46–59	169	42.1
	> 60	37	9.2
Marital status of respondent	Married	292	72.6
	Single	84	20.9
	Divorced	21	5.2
	Widowed	5	1.2
Religion of respondents	Muslim	127	31.6
	Orthodox	91	22.6
	Protestant	167	41.5
	Others	17	4.2
Resident	Urban	213	53
	Rural	189	47
Educational status	Unable to read and write	96	23.9
	Primary education	105	26.1
	Secondary education	135	33.6
	College and above	66	16.4
Purposed surgery	Major	341	84.8
	Minor	61	15.2
Previous surgery	Yes	14	3.5
	No	388	96.5

### Factors related with behavior, health status and clinical conditions

The majority of patients 396(98.5) did not use psycho active substance and 31 (7.7%) of the respondents had a history of chronic medical illness (Table 2).

**Table 2:** Clinical features that affect preoperative anxiety among adult patients undergoing elective surgery, at general hospital in Ethiopia, 2018 (*n* = 402)

Variables	Category	Frequency	%
Previous surgery	Yes	14	3.7%
	No	388	96.3%
Chronic medical illness	Yes	31	7.7%
	No	371	92.3%
Substance use	Yes	6	1.5%
	No	396	98.5%
Family mental illness	Yes	4	1%
	No	398	99%

**Fig. 1:**
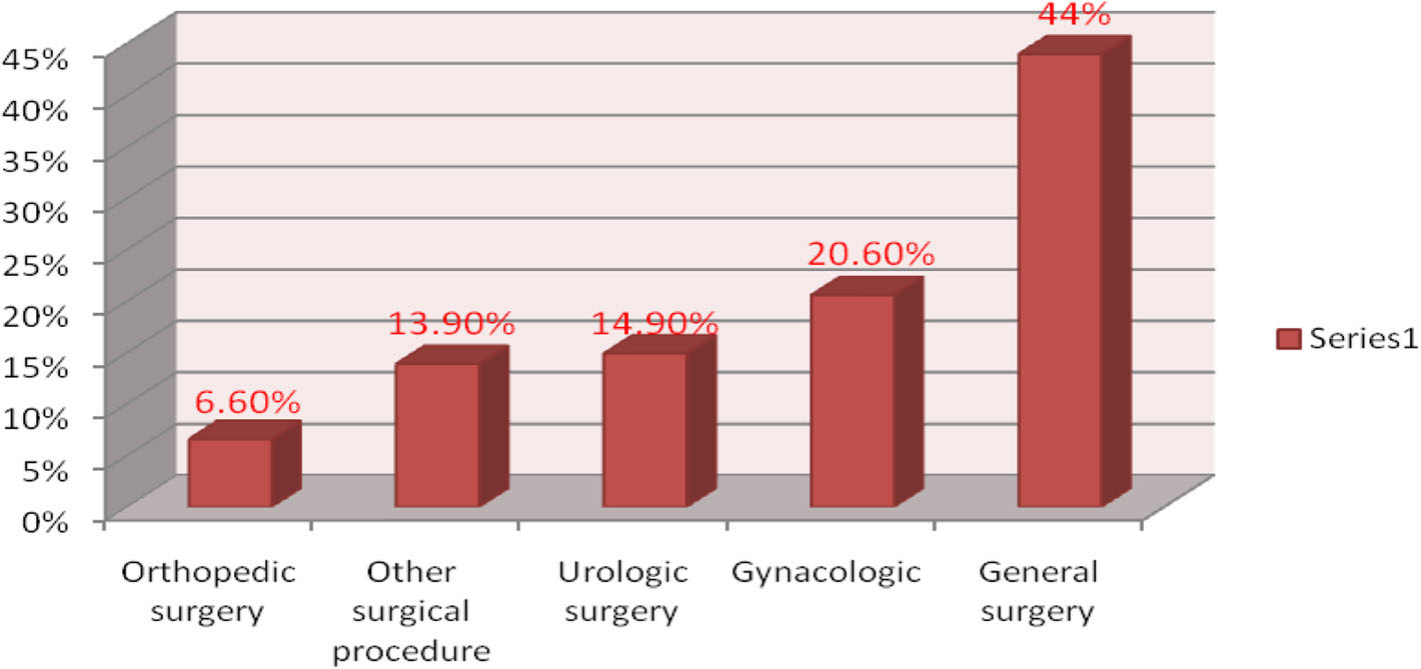
Type of surgery among patient who were undergoing elective surgery at general hospital in Ethiopia, 2018 (*n* = 402)

Majority 177(44%) of the procedure performed during study period was general surgery and 83(20.6%) were gynecologic surgery (Fig. 1).

### Prevalence and possible causes of preoperative anxiety

The prevalence of preoperative anxiety among study participants was 189 (47%). The patients were asked about the reasons they worried during the preoperative period and the most common reasons for preoperative anxiety were fear of death (83.1%), fear of complication (76.4%) and fear of unexpected result of operation (71.4%) (Table 3).

**Table 3:** Possible causes of preoperative anxiety among adult patients undergoing elective surgery at general hospital in Ethiopia, 2018 (*n* = 402)

Variables	Yes		No	
	Frequency	%	Frequency	%
Being received iv fluid	102	25.4	300	74.6
fear of death	334	83.1	68	16.9
Unexpected result of operation	287	71.4	115	28.6
Post-operative pain	245	60.9	157	39.1
Fear of anesthesia	264	65.7	138	34.3
Financial loss due to hospitalization	202	50.2	200	49.8
Fear of disability	217	54.0	185	46.0
Fear of complication	307	76.4	95	23.6
Harm from doctor or nurse mistake	116	28.9	286	71.1
Absence from work	130	32.3	272	67.7
Concern about family	205	51. 0	197	49.0
Cosmetics issues	142	45. 8	260	54.2
Need of blood transfusion	184	35.3	218	54.7
Unable to recover	225	56	177	44.0
Awareness during surgery	128	31.8	274	68.2
Information from previous hospital experiences	137	34.1	265	65.9
Fear of unknown	179	44.5	223	55.5

### Predictors of preoperative anxiety

During bivariate analysis factors with *p*-value less than 0.25 were entered in to multivariate logistic regression. Multivariate logistic regression revealed a significant association between preoperative anxiety and other independent variables with *p* value less than 0.05 (Table 4).

**Table 4:** Bivariate and multivariate logistic regression analyses of factors associayted with preoperative anxiety among adult patients undergoing elective surgery at general hospital in Ethiopia, 2018 (*n* = 402)

Variables		Anxiety status		COR (95% CI)	AOR (95%CI)	*P* value
		Yes	No			
Gender	Female	78	96	1	1	
	Male	111	117	1.16 (.78, 1.7)		
Age	18–30	54	81	1	1	
	31–45	64	82	1.11(.12, 9.80)		
	46–59	46	38	0.89(.18, 4.38)		
	> 60	25	12	0.90(.30,2.66)		
Residency	Urban	97	116	1	1	
	Rural	92	97	1.13(.76,1.67)		
Marital status	Single	28	56	1	1	
	Married	147	145	2.02(1.22,3.37)	1.44(.77, 2.7)	0.25
	Divorced	11	1 0	2.20(.83,5.79)	1.57(0.53,4.7)	0.42
	Widowed	3	2	3(0.47,19)	2.26(0.22, 23)	0.48
Proposed surgery	Major	160	181	1	1	
	Minor	29	32	0.975(.56, 1.68)		
Social support	Poor	35	22	1	1	
	Moderate	123	84	5.49(2.8, 10.7)	0.84(0.44, 1.6)	0.616
	Strong	31	107	5.05(3.10, 8.2)	0.16(.07, .34)	0.00*
Educational status	Illiterate	59	37	1		1
	Primary	47	58	2.45(1.29, 4.6)	0.58(.31, 1.)	0.89
	Secondary	57	78	1.24(.66, 2.33)	0.69(.36, 1.1)	0.25
	>college	26	40	1.12(.61, 2.04)	0.59(.28, 1.21)	0.15
Surgical procedure	General	65	112	.72(.39, 1.32)	0.74(.37, 1.47)	0.39
	Gynecology	45	38	1.46(.74, 2.90)	1.12(.5, 2.45)	0.77
	Orthopedic	13	13	1.24(.48, 3.14)	2.8(1.18,6.9)	0.20
	Urologic	41	19	2.67(1.25,5.70)	1.17(.42. 3.2)	0.78
	Other	25	31	1		
Previous surgery	Yes	12	201	0.27(0.075, .97)		
	No	3	186	1		
Chronic medical illness	Yes	17	196	1.08(0.51, 2.26)		
	No	14	175	1		
Substance use	Yes	5	208	5.4(.06, 45.68)	9.2(.95, 88.8)	.055
	No	1	188	1		1
Family mental illness	Yes	3	210	4(.12, 128.5)		
	No	1	188	1		

*variable with *P* value less than 0.05(Significantly association)

The multivariate analysis revealed a significant association was found between preoperative anxiety and the possible causes of anxiety, which are; fear of unknown, fear of anesthesia, fear of unexpected result of operation, need of blood transfusion, fear of harm from doctors or nurse mistake, and concern about family (Table 5).

**Table 5:** Bivariate and multivariate logistic regression analyses of association between possible causes of anxiety with preoperative anxiety among adult patients undergoing elective surgery, at general hospital in Ethiopia, 2018 (*n* = 402)

Variables		Anxiety status		COR(95%CI)	AOR(95%CI)	*P*-value
		Yes	No			
Being received iv fluid	Yes	52	161	1.01(.57,1.79)		
	No	50	139	1		1
Fear of death	Yes	171	42	1.3(.71,2.49)		
	No	163	26	1		1
Unexpected result	Yes	127	86	2.72(1.48,5.02)	3.03(1.7, 5.19)	0.001*
	No	160	29	1		
Post-operative pain	yes	109	136	1.41(.78, 2.5)		
	No	104	53	1		1
Fear of anesthesia	Yes	122	91	1.28(.73, 2.26)		
	No	142	47			
Financial loss	yes	98	115	1		1
	No	104	85	0.75(.43, 1.29)		
Fear of disability	Yes	97	116	1		1
	No	120	69	0.83(.45, 1.53)		
Fear of complication	Yes	146	67	1.46(.80, .26)		
	No	161	28	1		1
Fear of harm from doctors /nurse mistake	Yes	71	42	4.59(2.43, 8.66)	5.03(2.85, 8.8)	0.001*
	No	45	144	1		1
Fear of Absence from work	Yes	66	147	1		1
	No	64	125	1.20(.65, 2.18)		
Family concern	Yes	88	125	1		1
	No	117	72	1.07(.62, 1.83)		
Need of blood transfusion	Yes	72	141	2.5(1.47, 4.51)	2.7(1.65, 4.62)	0.001*
	No	112	77	1		1
Cosmetic issues	Yes	67	146	1		1
	No	75	114	1.26(.72, 2.19)		
Unable to recover	Yes	91	122	2.95(1.63, 5.34)	2.96(1.8, 4.87)	0.000*
	No	134	55	1		1
Awareness during surgery	Yes	64	149	0.90(.48, 1.69)		
	No	64	125	1		1
Information from prev. Surgery	Yes	64	149	1.16(.61, 2.20)		
	No	73	116	1		1
Fear of unknown	Yes	93	120	1.07(.63, 1.8)		

## Discussion

The present study found that 47% of the subjects who were waiting for elective surgery experienced preoperative anxiety as suggested by STAI score of above 44. The finding in current study finding for preoperative anxiety was higher than the studies conducted at Addis Ababa, Ethiopia 39.8% [15]. The reason for the above difference might be due to that the patient had less access to information regarding their surgery compared to our study area. On the other hand the finding of this study on prevalence of preoperative anxiety was lower than a study conducted at Pakistan 62% [14], 76.7% at Sri Lanka [12], Tunisia (67.5%) [6], 70.3% at Jimma [11], and 59.6% at Gondar [8]. The variation of the current study can be explained by inclusion of younger age participants and use of different assessment tool in those studies.

This study revealed that variables like using psycho active substances, having strong social support, unexpected result of operation, fear of harm from doctor or nurse mistake, need of blood transfusion, and unable to recover were found to be statistically significant for preoperative anxiety.

Substance user have marginal significant association with preoperative anxiety, which is those who have history of current substance use were nine times more likely to develop preoperative anxiety (AOR = 9.20, CI = (0.95, 88.76) compared with their counterpart. This may be due to that they were substance addicted and experienced hopelessness and not willing to get health related advices or being careless. The study was supported by previous study [8].

Participants who had strong social support (AOR = 0.16, CI = (0.07, 0.34) were 84% less likely to become anxious during preoperative period compared with those who have poor social support. This might be due to close relationship have high affinity on reduction of preoperative anxiety. The findings in the current study was supported by study conducted in Addis Ababa [15], and Thailand [5], showed that preoperative anxiety among those who have poor social support was higher compared to those who have strong social support social support.

We found that respondents with fear of need of blood product receiving were two times more likely to develop preoperative anxiety (AOR = 2.76, CI = (1.65, 4.62) when compared with those who don’t need. This might be due to unable to find compatible blood group or financial loss to get blood.

Participants having fear of unexpected result of operation were three times more likely to develop preoperative anxiety with (AOR = 3.03, CI = (1.73, 5.19) compared with those haven’t. The reason behind is that participants thought death and disability may occur following operation. Those having fear of harm from doctors or nurse mistake were five times more likely to became anxious with (AOR = 5.03, CI = 2.85, 8.86) in our finding. This study was supported by study conducted in Addis Ababa with (AOR = 2.24, CI = 1.05, 1.91) [15]. Patients having fear of unable to recover were two times more likely to develop preoperative anxiety with (AOR = 2.96, CI = (1.81, 4.87) those who don’t have fear. This study was in line with the study conducted in Gondar (AOR = 1.31, CI = (0.62, 2.77)) [8]. The reason behind is that participants having thought of death and disability following operation might result in developing preoperative anxiety.

The main limitation of the study is: since the study design is cross-sectional, it was difficult to establish cause and effect relationship.

## Conclusion

In the current study the prevalence of preoperative anxiety was high (47.0%). Having strong social support, unexpected result of operation, fear of harm from doctor or nurse mistake, need of blood transfusion, and fear of unable to recover were found to be statistically significant for preoperative anxiety. Patients need to be assessed regularly for anxiety during the preoperative visit and appropriate anxiety reduction methods should be introduced.

## Additional file

Additional file 1: Data collection tool and informed consent on assessment of preoperative anxiety among adult patients undergoing elective surgery: a prospective survey at general hospital in Ethiopia, 2018 (*n* = 402). (DOCX 38 kb)

## Abbreviations

AOR: Adjusted Odds Ratio
CBE: Community based education
CI: Confidence Interval
COR: Crudes Odds Ratio
ETB: Ethiopian birr
GYN: Gynecological
POA: Preoperative Anxiety
SNNPRS: South Nation, Nationalities, and Peoples State
STAI: State Trait Anxiety Inventory
