# Supplementary material for: Preoperative anxiety among adult patients undergoing elective surgery: a prospective survey at a general hospital in Ethiopia
Source: Patient Saf Surg. 2019 Apr 8;13:18. doi: 10.1186/s13037-019-0198-0 (PMC6454677; doi:10.1186/s13037-019-0198-0)
Supplement: Supplementary file 1 — Data collection tool and informed consent on assessment of preoperative anxiety among adult patients undergoing elective surgery: a prospective survey at general hospital in Ethiopia, 2018 (n = 402). (DOCX 38 kb) [file 13037_2019_198_MOESM1_ESM.docx]

**Additional file 1:** Data collection tool and informed consent on assessment of Preoperative anxiety among adult patients undergoing elective surgery: a prospective survey at general hospital in Ethiopia, 2018 (**n=402**).

**Informed Consent**

Good morning/afternoon!

My name is _________________, I am working as data collector in a study conducted by Hawassa University, college of medicine and health sciences. This questionnaire is prepared to conduct a study **“**Preoperative anxiety among adult patients undergoing elective surgery: a prospective survey at general hospital in Ethiopia”. You are selected and included in the study as part of the sample population to complete the questionnaire designed by the researchers. The data you will provide is very helpful to give important comments that will help to strengthen and improve mental health service for patients undergoing elective surgery. The information obtained in this study will be used only for research purposes. Your name will not be put in the format. Any information obtained will be kept strictly confidential and will not be exposed to any other body in connection to your name. Your participation is voluntary and you are not obliged to answer any questions you don't want.

But your honest participation will contribute a lot to generate information and to come up with important findings. The interview will take about 20 minutes. If you have any questions regarding the study, you can contact the following individual with their address.

Do you want to participate in the study?

1. Yes
2. No

**PART I: Socio-Demographic and Clinical Characteristics**

| No | Question | Answer | | | | | |
| --- | --- | --- | --- | --- | --- | --- | --- |
| 101 | Age in Years | | _________________ | | | | |
| 102 | Gender | 1. Male 2. Female | | | | | |
| 103 | Religion | 1. Orthodox 2. Protestant 3. Muslim 4. Other ( Specify)___________ | | | | | |
| 104 | Educational status | 1. Unable to read and write 2. Primary Education 3. Secondary Education 4. College and above | | | | | |
| 105 | Ethnicity | 1. Oromo 2. Amhara 3. Sidama 4. Wolaita 5. Gedeo 6. Other Specify_____________ | | | | | |
| 106 | Marital Status | 1. Single 2. Married 3. Divorced 4. Widow | | | | | |
| 107 | Occupation | 1. Government Employed 2. Private employed 3. Merchant 4. Daily labor 5. House Wife 6. Jobless 7. Other, Specify____________ | | | | | |
| 108 | Residence | | | 1. Urban 2) Rural | | | |
| 109 | Purposed surgery | | | 1. Major 2) Minor | | | |
| 110 | Surgical procedure | | | 1. General 2. Gynecological 3. Orthopedics 4. Urology 5. Other, specify_________________ | | | |
| 111 | Do you have follow up on chronic medical illness like Ca, DM, HTN, HIV/AIDS, CVDs…? | | | | | | 1. Yes 2. No |
| 112 | Previous Surgery | | | | | | 1. Yes 2) No |
| 113 | Is there any one who is diagnosed with mental illness in your familly? | | | | | | 1. Yes 2. No |
| 114 | Do yo have history of any type ofdiagnosed mental illness previously? | | | | | | 1. Yes 2. No |
| 115 | Do you use the any type of psychoactive substance over the last 3 months? | | | | | 1. Yes 2. No | |
| 116 | If yes for question number 118, what type of substance did you use? | | | | 1. Khat 2. Tobacco 3. Alcohol 4. Other(specify)__________ | | |

| Q. no | Questionnaire | Categories/alternative/ |
| --- | --- | --- |
| Q301 | How many people are you so close to that you can count on them if you have great personal problems? | 1. None 2. 1-2 3. 3-5 4. 5+ |
| Q302 | How much interest and concern do people show in what you do? | 1. None 2. Little 3. Uncertain 4. Some 5. A lot |
| Q303 | How easy is it to get practical help from neighbors if you should need it? | 1. Very difficult 2. Difficult 3. Possible 4. Easy 5. Very easy |

**Section II: Oslo Social Support Assessment to assess social support**

**PART III: Possible Causes of Preoperative Anxiety**

| S.No | variable | 1. Yes | 1. No |
| --- | --- | --- | --- |
| 1 | Being received IV fluid | 1 | 0 |
| 2 | Fear of Death | 1 | 0 |
| 3 | Unexpected results of operation | 1 | 0 |
| 4 | Post-operative pain | 1 | 0 |
| 5 | Fear of Anesthesia | 1 | 0 |
| 6 | Fear of death | 1 | 0 |
| 7 | Financial loss due to hospitalization | 1 | 0 |
| 8 | Fear of physical disability | 1 | 0 |
| 9 | Fear of complications | 1 | 0 |
| 10 | Harm from doctor or nurse mistake | 1 | 0 |
| 11 | Absence from work | 1 | 0 |
| 12 | Concern about family | 1 | 0 |
| 13 | Need of blood transfusion | 1 | 0 |
| 14 | Cosmetic issues | 1 | 0 |
| 15 | Unable to recover | 1 | 0 |
| 16 | Awareness during surgery | 1 | 0 |
| 17 | Information from previous negative hospital experiences | 1 | 0 |
| 18 | Fear of unknown | 1 | 0 |

**Part IV: State-Trait Anxiety Inventory (Questionnaires): STAI Form Y-1**

DIRECTIONS: A number of statements which people have used to describe themselves are given below. Read each statement and then write the number in the blank at the end of the statement that indicates how you feel right now‚ that is‚ at this moment. There is no right or wrong answers. Do not spend too much time on any one statement but give the answer which seems to describe your present feelings best.

| S.No | Variable | Not at all | Some What | Moderately | Very much so |
| --- | --- | --- | --- | --- | --- |
| 1 | I feel calm | 1 | 2 | 3 | 4 |
| 2 | I feel secure | 1 | 2 | 3 | 4 |
| 3 | I am tense | 1 | 2 | 3 | 4 |
| 4 | I feel Strained | 1 | 2 | 3 | 4 |
| 5 | I feel at ease | 1 | 2 | 3 | 4 |
| 6 | I feel upset | 1 | 2 | 3 | 4 |
| 7 | I am presently worrying over possible misfortunes | 1 | 2 | 3 | 4 |
| 8 | I feel satisfied | 1 | 2 | 3 | 4 |
| 9 | I feel frightened | 1 | 2 | 3 | 4 |
| 10 | I feel comfortable | 1 | 2 | 3 | 4 |
| 11 | I feel self confident | 1 | 2 | 3 | 4 |
| 12 | I feel nervous | 1 | 2 | 3 | 4 |
| 13 | I am Jittery | 1 | 2 | 3 | 4 |
| 14 | I feel indecisive | 1 | 2 | 3 | 4 |
| 15 | I am relaxed | 1 | 2 | 3 | 4 |
| 16 | I feel content | 1 | 2 | 3 | 4 |
| 17 | I am worried | 1 | 2 | 3 | 4 |
| 18 | I feel confused | 1 | 2 | 3 | 4 |
| 19 | I feel steady | 1 | 2 | 3 | 4 |
| 20 | I feel pleasant | 1 | 2 | 3 | 4 |

**STAI form Y-2**

DIRECTONS: A number of statements which people have used to describe themselves are given below. Read each statement and then write the number in the blank at the end of the statement that indicates how you generally feel. There is no right or wrong answer. Do not spend too much time on any one statement but give the answer which seems to describe how you generally feel.

| S.No | Variable | Almost never | Some times | Often | Almost always |
| --- | --- | --- | --- | --- | --- |
| 21 | I feel pleasant | 1 | 2 | 3 | 4 |
| 22 | I feel nervous and restless | 1 | 2 | 3 | 4 |
| 23 | I feel satisfied with myself | 1 | 2 | 3 | 4 |
| 24 | I wish I could be as happy as others seem to be | 1 | 2 | 3 | 4 |
| 25 | I feel like a failure | 1 | 2 | 3 | 4 |
| 26 | I feel rested | 1 | 2 | 3 | 4 |
| 27 | I am calm, cool, and collected | 1 | 2 | 3 | 4 |
| 28 | I feel that difficulties are piling up so that I cannot overcome them | 1 | 2 | 3 | 4 |
| 29 | I worry too much over something that really doesn’t matter | 1 | 2 | 3 | 4 |
| 30 | I am happy | 1 | 2 | 3 | 4 |
| 31 | I have disturbing thoughts | 1 | 2 | 3 | 4 |
| 32 | I lack self confidence | 1 | 2 | 3 | 4 |
| 33 | I feel secure | 1 | 2 | 3 | 4 |
| 34 | I make decision easily | 1 | 2 | 3 | 4 |
| 35 | I feel inadequate | 1 | 2 | 3 | 4 |
| 36 | I am content | 1 | 2 | 3 | 4 |
| 37 | Some unimportant thoughts runs through my mind and bothers me | 1 | 2 | 3 | 4 |
| 38 | I take disappointments so keenly that I can’t put them out of my mind | 1 | 2 | 3 | 4 |
| 39 | I am a steady person | 1 | 2 | 3 | 4 |
| 40 | I get in a state of tension or turmoil as I think over my recent concerns and interests | 1 | 2 | 3 | 4 |
